# DNA studies are necessary for accurate patient diagnosis in compound heterozygosity for Hb Adana (HBA2:c.179>A) with deletional or nondeletional α-thalassaemia

**DOI:** 10.1038/srep26994

**Published:** 2016-06-08

**Authors:** Jin Ai Mary Anne Tan, Siew Leng Kho, Chin Fang Ngim, Kek Heng Chua, Ai Sim Goh, Seoh Leng Yeoh, Elizabeth George

**Affiliations:** 1 Department of Biomedical Science, Faculty of Medicine, University of Malaya, Kuala Lumpur, Malaysia; 2 Department of Paediatrics, School of Medicine and Health Sciences, Monash University Sunway Campus, Selangor, Malaysia; 3 Department of Paediatrics, Hospital Pulau Pinang, Jalan Residensi, Penang, Malaysia; 4 Assunta Hospital, Petaling Jaya, Selangor, Malaysia

## Abstract

Haemoglobin (Hb) Adana (HBA2:c.179>A) interacts with deletional and nondeletional α-thalassaemia mutations to produce HbH disorders with varying clinical manifestations from asymptomatic to severe anaemia with significant hepatosplenomegaly. Hb Adana carriers are generally asymptomatic and haemoglobin subtyping is unable to detect this highly unstable α-haemoglobin variant. This study identified 13 patients with compound heterozygosity for Hb Adana with either the 3.7 kb gene deletion (-α^3.7^), Hb Constant Spring (HbCS) (HBA2:c.427T>C) or Hb Paksé (HBA2:429A>T). Multiplex Amplification Refractory Mutation System was used for the detection of five deletional and six nondeletional α-thalassaemia mutations. Duplex-PCR was used to confirm Hb Paksé and HbCS. Results showed 84.6% of the Hb Adana patients were Malays. Using DNA studies, compound heterozygosity for Hb Adana and HbCS (α^codon 59^α/α^CS^α) was confirmed in 11 patients. A novel point in this investigation was that DNA studies confirmed Hb Paksé for the first time in a Malaysian patient (α^codon 59^α/α^Paksé^α) after nine years of being misdiagnosis with Hb Adana and HbCS (α^codon 59^α/α^CS^α). Thus, the reliance on haematology studies and Hb subtyping to detect Hb variants is inadequate in countries where thalassaemia is prevalent and caused by a wide spectrum of mutations.

Thalassaemia, a genetic haemoglobin (Hb) disorder commonly present in the Mediterranean, North Africa, Middle East, India and Southeast Asia, has become a worldwide public health problem^1^,^2^. The recognition of thalassaemia as a global disorder has led to the implementation of public education and newborn screening programs in countries where thalassaemia was previously a rare disorder. The most severe form of α-thalassaemia is Hb Bart’s hydrops foetalis syndrome, where the foetus usually dies *in-utero* or soon after birth. HbH disease is a non-fatal α-thalassaemia that produces moderate to severe α-thalassaemia with more clinical manifestations of severe haemolytic anaemia and hepatosplenomegaly^3^. HbH disease was once prevalent mainly in Asia, Middle East and the Mediterranean, but the patterns of the disorder are now different as a result of changing demographics in intermarriages and population migration. In a study in California, 56% of HbH disease detected through newborn screening was reported in babies with Asian, Hispanic and African-American parents living in the USA^4^.

Deletional HbH disease caused by the removal of three of the four α-globin genes is more common and patients show mild and homogenous clinical manifestations^3^. However, nondeletional α-thalassaemia mutations are also involved in HbH disease and are called nondeletional HbH disease. This disorder is more serious and causes significant hepatosplenomegaly and severe anaemia which requires regular blood transfusions^3^,^5^,^6^. The most common nondeletional α-thalassaemia mutations in Southeast Asia are Hb Constant Spring (HbCS) (HBA2:c.427T>C), Hb Adana (HBA2:c.179G>A), Hb Quong Sze (HbQS) (HBA2:c.377T>C), Hb Paksé (HBA2:429A>T) and Hb Suan Dok (HBA2:c.329T>G)^7^,^8^,^9^,^10^. In Malaysia, HbCS is most common in the indigenous populations followed by the Malays and to a lesser frequency in the Chinese^11^,^12^. Hb Adana was reported in the Malaysian Malays, Chinese and indigenous populations, HbQS was observed at a much lesser frequency and Hb Paksé has not been reported^12^,^13^. The first reported case of Hb Adana in Malaysia was in a 52-year-old patient with α-thalassaemia intermedia^14^. Hb Adana and HbCS carriers are generally asymptomatic with normal Hb levels. In addition, their red cell indices are similar to α^+^-thalassaemia carriers with a single gene deletion, thus silent carriers are common. Confirmation of Hb Adana is a diagnostic predicament as this haemoglobin variant is highly unstable and no product is observed during routine haematology tests. Thus, patients may remain undiagnosed until DNA studies are carried out.

Using DNA studies, we report here one case of Hb Adana and 3.7 kb α-globin gene deletion (NG_000006.1:g.34164_37967del3804), 11 cases of severe α-thalassaemia intermedia patients with compound heterozygosity for Hb Adana and HbCS, and one rare case of Hb Adana and Hb Paksé. These 13 cases were accurately identified only after DNA studies were carried out.

## Results

Routine haematology studies were carried out for all patients, their parents and affected siblings. Molecular tests showed the patients to be negative for deletional α-thalassaemia: -α^4.2^, –^SEA^ (NG_000006.1:g.26264_45564del19301), –^FIL^ (NG_000006.1:g.11684_43534), –^THAI^ (NG_000006.1:g.10664_44164del33501), and the nondeletional α-thalassaemia mutations: initiation codon (HBA2:c.2delT), codon 30 (HBA2:c.91_93delGAG), codon 35 (HBA2:c.106T>C) and HbQS. DNA sequencing showed the mutation for Hb Adana in all the 13 patients to be present in codon 59 in the α2-globin gene (Supplementary Figure 1A,B).

### Compound heterozygosity for Hb Adana and 3.7 α-globin gene deletion (α^codon 59^α/-α^3.7^)

Initial DNA studies detected only the 3.7 α-globin gene deletion (-α^3.7^) in a Malay patient when she presented with thalassaemia intermedia at 30 years of age (Table 1, P1). Repeat DNA studies carried out during a follow up when the patient was 42 years-old confirmed the patient to be compound heterozygous for Hb Adana and 3.7 kb α-globin gene deletion (α^codon 59^α/-α^3.7^).

### Compound heterozygosity for Hb Adana and Hb Constant Spring (α^codon 59^α/α^CS^α)

DNA studies showed compound heterozygosity for Hb Adana and HbCS in 9 Malay patients, one patient with Malay-Indian ancestry and one Chinese patient (Table 1, P2–P12). During initial thalassaemia screening, Hb Adana was not detected by High Performance Liquid Chromatography (HPLC) in all the 11 patients and their parents. The parents with HbCS showed normal blood films and their H-inclusion tests were negative. HbCS was confirmed by HPLC in only 8 of the 11 cases as HbCS is an unstable α-globin chain variant comprising of only 1–2% of total haemoglobin, and may be missed during routine screening. Diagnosis of compound heterozygosity for Hb Adana and HbCS (α^codon 59^α/α^CS^α) was confirmed only after DNA studies were carried out.

### Compound heterozygosity for Hb Adana and Hb Paksé (α^codon 59^α/α^Paksé^α)

The patient presented with pallor and hepatosplenomegaly at one-year-old with Hb of 4.77 g/dL (Table 1, P13). He required at least two monthly transfusions and was subsequently started on hyper-transfusion regimen (every four to six weeks) at four years of age due to poor growth. Haematology results from the patient’s father showed normochromic, normocytic red cells, negative H-test, normal Hb A_2_ and Hb F, and DNA studies confirmed Hb Adana. The patient’s mother was confirmed with HbCS by HPLC and initial DNA studies also showed HbCS. However, her haematology results were too severe for heterozygous HbCS (Hb: 9.8 g/dL; Mean Cell Volume (MCV): 77.2; Mean Cell Haemoglobin (MCH): 23.7; blood film showed hypochromia and anisocytosis with target cells). Repeat DNA studies for other α-globin gene mutations were requested and his mother was then confirmed to be compound heterozygous for HbCS and Hb Paksé (α^CS^α/α^Paksé^α). Compound heterozygosity for Hb Adana and Hb Paksé (α^codon 59^α/α^Paksé^α) was confirmed in the patient only after nine years of follow up when DNA studies were performed.

## Discussion

Compound heterozygosity for Hb Adana with deletional and non-deletional mutations have been reported in Malaysian patients with varying clinical phenotypes^12^,^13^,^15^. In a Malaysian patient with severe anaemia, but without family history of thalassaemia, HPLC detected Hb A, Hb A_2_, Hb F and a pre-run peak of Hb Bart’s with absence of abnormal haemoglobins^14^. Although this patient showed all the clinical features of α-thalassaemia intermedia, he had a negative H-inclusion test. Compound heterozygosity (α^codon 59^α/-α^3.7^) was confirmed only after DNA studies were carried out. In this study, P1 possessed the same genotype as the above reported case, except that her Hb Adana was present in the α2-gene. P1 presented at 30-years-old with hypochromic and microcytic anaemia (Table 1, P1). She was non-transfusion dependent and required monthly transfusions only during pregnancy. At 41 years old, she started regular transfusions at four to six weekly intervals for symptomatic anaemia. Compound heterozygosity for Hb Adana and α-globin gene deletions were also reported in two Albanian patients with thalassaemia intermedia, mild to moderate anaemia and isolated cases with severe anaemia in Indonesian patients, and a very mild phenotype in a Greek patient^5^,^6^,^16^. In addition, compound heterozygosity for Hb Adana (α1-gene) and a large 20.5 kb deletion (NG_000006.1:g.15164_37864del22701) (α^codon 59^α/-α^20.5^) was reported in two patients with severe HbH disease^17^. These reports confirm the wide variety of clinical phenotypes exhibited by patients with compound heterozygosity for Hb Adana and deletional α-thalassaemia. It is difficult to associate genotypes with clinical manifestations as different clinical phenotypes (initial presentation of disorder, first transfusion and duration between transfusions) can be expressed even within the same family.

Using DNA studies, this study also detected 11 patients with compound heterozygosity for Hb Adana and the nondeletional haemoglobin variant HbCS (α^codon 59^α/α^CS^α) (Table 1, P2–P12). Hb Adana and HbCS carriers are usually asymptomatic with normal haemoglobin levels and their blood indices are within normal limits or show only slight reductions in MCV and MCH. In addition, HPLC for Hb subtypes does not detect Hb Adana and occasionally misses HbCS as both are unstable haemoglobins produced in small quantities. These problems were similarly observed in the parents of our patients. Four of these patients have siblings with the same genotype and all patients and siblings showed severe α-thalassaemia (Table 1, P7–P10). Results from this study are similar to other reports where compound heterozygosity for Hb Adana with nondeletional mutations produce HbH disease with more severe anaemia, earlier presentation and possible blood transfusion requirements when compared with the less severe disorders of deletional HbH disease^3^,^6^,^18^. In a case series where two siblings with compound heterozygosity for Hb Adana and HbCS were compared with another two siblings with Hb Adana and a 3.7 kb α-globin gene deletion, the siblings with compound heterozygosity for Hb Adana and the nondeletional mutation HbCS produced more severe clinical manifestations^15^.

This study reports a rare case of compound heterozygosity for Hb Adana and Hb Paksé (Table 1, P13). Hb Paksé was misdiagnosed as HbCS in about 15% of patients in Thailand as both Hb variants are produced at low levels and have similar migration rates during Hb electrophoresis^9^. In this study, the patient’s mother was misdiagnosed twice as heterozygous for HbCS (α^CS^α/αα) by HPLC. Hb Paksé was not detected in her first DNA study as the assay did not contain primers for Hb Paksé. Her unusual haematology results warranted further DNA studies and compound heterozygosity for HbCS and Hb Paksé (α^codon 59^α/α^Paksé^α) was finally confirmed. Routine haematology tests and Hb subtyping were performed twice for the patient’s father and he was diagnosed to be normal on both occasions. He was confirmed to be heterozygous for Hb Adana (α^codon 59^α/αα) only after DNA studies. Our patient with compound heterozygosity for Hb Adana and Hb Paksé showed severe α-thalassaemia intermedia requiring a hyper-transfusion regimen. In a published report of patients with compound heterozygosity for HbE/HbCS and HbE/Hb Paksé, these combinations were not associated with severe anaemia^19^. Thus, this again indicates the wide spectrum of clinical manifestations shown by interactions between different Hb variants.

Results from this study indicate that Hb Adana is more prevalent in the Malaysian Malays as 84.6% (11/13) of the patients were from this ethnic group and one patient was of Malay-Indian ancestry. This observation is in concordance with other published reports of Hb Adana in Malaysia^12^,^13^. The reliance on routine haematology studies and Hb subtype analysis to detect Hb Adana may not be sufficiently accurate in populations with different globin gene mutations. This is further supported in a publication where α2 Hb Adana was confirmed only after Multiplex-ARMS and DNA sequencing were carried out in a patient with compound heterozygosity for Haemoglobin Q-Thailand and Hb Adana^20^.

Thalassemia is a public health problem in Malaysia and identification of the wide spectrum of globin gene mutations is challenging due to the multiracial population. In this study, analysis of results from the patients’ case notes showed that 11 patients had been previously misdiagnosed using haematology studies, Hb subtyping and molecular analysis. Additionally, 2 patients (P5 and P9) whose case notes were not available and who were previously diagnosed with HbH disease with HbCS were only accurately diagnosed with Hb Adana and HbCS in this study. Thus, the present study highlights the importance of complete molecular studies in patients where specific red cell indices and abnormal haemoglobins are absent during routine haematological tests.

This study will also contribute to more effective diagnosis of HbH disorders using the various molecular methods presented. The outlook for accurate diagnosis for Malaysian patients with α-thalassaemia intermedia with normal Hb subtypes, presence of Hb Bart’s but negative H-inclusion tests will be complete DNA studies of deletional and non-deletional α-thalassaemia mutations.

Future work should involve the reassessment of patients who were previously diagnosed with HbCS using only Hb subtyping as there may be a possibility of misdiagnosis^9^. Furthermore, as Malaysia and Thailand share borders and intermarriages between the two populations do take place, Hb Paksé should be added in the panel for DNA studies in Malaysia.

## Methods

### Patients and haematological tests

Blood samples from 12 patients with severe α-thalassaemia intermedia and one patient with α-thalassaemia intermedia were sent to University of Malaya for DNA studies (Table 1). The ethnic distributions of the patients are Malays (11), Malay-Indian (1) and Chinese (1). The ages of the patients ranged from six months to 42 years. Blood samples were also collected from siblings of four of the patients (Table 1, P7–P10) who also showed severe α-thalassaemia intermedia. Routine haematology tests were conducted by established techniques. Complete blood cell count was measured on EDTA-anticoagulated blood samples using an automated blood counter (Sysmex Corporation), red cell morphology was examined on stained peripheral blood films (Romanowsky-Giemsa stain) and H-inclusion test was carried out. The blood samples were screened for thalassaemia by HPLC BioRad Variant II (BioRad Laboratories, Hercules, CA, USA) using the β-thalassaemia short program.

### DNA analysis for deletional α-thalassaemia

The five common deletions causing α-thalassaemia in Southeast Asia: -α^3.7^, -α^4.2^, –^SEA^, –^FIL^, –^THAI^ were characterised using an adapted multiplex-PCR^21^ (Supplementary Table 1). Hot Start *Taq* DNA polymerase (Qiagen HotStarTaq *Plus* DNA Polymerase, Hilden, Germany) was used for DNA amplification. The modified PCR conditions for DNA amplification were as follows: initial denaturation at 96 °C for 5 minutes followed by 30 cycles at 98 °C for 45 seconds, 64 °C for 1 minute 30 seconds and 72 °C for 2 minutes 15 seconds with a final extension of 72 °C for 5 minutes.

### Multiplex Amplification Refractory Mutation System for nondeletional α-mutations

Six nondeletional α-globin gene mutations were characterised using a modified Multiplex Amplification Refractory Mutation System (ARMS) assay^22^ (Supplementary Table 2). The mutations characterised were as follows: initiation codon (ATG → A-G), codon 30 (ΔGAG), codon 35(TCC → CCC), Hb Adana, HbCS and HbQS. The PCR cycling conditions were initial denaturation at 94 °C for 10 minutes followed by 30 cycles at 94 °C for 40 seconds, 62 °C for 20 seconds and 72 °C for 3 minutes with a final extension of 72 °C for 5 minutes.

### Duplex-PCR for Hb Paksé and Hb Constant Spring

DNA amplification for Hb Paksé and HbCS was carried out using primer sequences in a modified duplex-PCR^23^ (Supplementary Table 3). Hot Start *Taq* DNA polymerase (Qiagen HotStarTaq *Plus*) was used for DNA amplification. The modified PCR conditions for optimal DNA amplification were initial denaturation at 94 °C for 5 minutes followed by 30 cycles at 94 °C for 1 minute and 62 °C for 1 minute 30 seconds. Amplified DNA was electrophoresed in 1.5% (w/v) agarose gels with ethidium bromide and visualised using a UV illuminator.

### DNA sequencing for Hb Adana

DNA sequencing for Hb Adana was carried out using published primer sequences^24^ (Supplementary Table 4). Sequencing was carried out using the ABI PRISM BigDye Primer Sequencing Ready Reaction Kit (Applied Biosystems, California, USA) according to the manufacturer’s instructions.

### Ethics statement

This study was approved by the Medical Ethics Committee of University Malaya Medical Centre. All methods used in this study were in accordance with the approved guidelines by University Malaya Medical Centre. Patients who were under the care of their consultant physicians (or their parent/legal guardian) requested molecular characterisation of their condition. Consultant physicians explained the process, and obtained written and oral permission to carry out the molecular work. Blood was taken only after securing patient consent (or that of parent/legal guardian). Patients (or their parent/legal guardian) were informed that anonymised data (i.e. patient name redacted) would be published.

## Additional Information

**How to cite this article**: Tan, J. A. M. A. *et al.* DNA studies are necessary for accurate patient diagnosis in compound heterozygosity for Hb Adana (HBA2:c.179>A) with deletional or nondeletional α-thalassaemia. *Sci. Rep.* **6**, 26994; doi: 10.1038/srep26994 (2016).

## Supplementary Material

Supplementary Information

## Figures and Tables

**Table 1:** Demographic data and clinical manifestation of patients with compound heterozygosity for Hb Adana and deletional (-α^3.7^) or nondeletional α-thalassaemia.

	Sex-Age (years)	Ethnic Group	Clinical Manifestation
P1	F-42	Malay	Presented at 30-year-old with hypochromic and microcytic anaemia. No transfusion was required except during pregnancy. Started on 3–4 monthly transfusions at 41-year-old for symptomatic anaemia.
P2	F-5	Malay	Presented at 2 years suggestive of α-thalassaemia and unusual haemoglobinopathy. Presented again at 5 years with chronic anaemia and significant hepatosplenomegaly. Patient on hyper-transfusion regimen.
P3	F-0.5	Malay	Presented at 5 months with background history of conjugated bilirubin, raised liver enzyme and neonatal hepatitis. Peripheral blood film suggestive of haemolysis and enzymopathy.
P4	F-25	Malay	Transfused since 6-year-old.
P5	M-15	Malay	Case notes were not available.
P6	M-13	Malay	Transfused since 5-month-old.
P7^*^	F-11	Malay	Patient showed haemolytic anaemia with frontal bossing, tinge of jaundice, maxillary hyperplasia and hepatosplenomegaly.
P8^*^	F-9	Malay	Presented at 1 year 7 months and diagnosed with HbH disease with HbCS. Presented again at 9 years with anaemia and hepatosplenomegaly.
P9^*^	F-12	Malay	Case notes were not available.
P10^*^	M-15	Malay	Presented at 3 years old with Hb between 6 to 7.8 g/dL. Noted fall off in height centiles at 14 years old with no puberty features and increasing spleen size (9 cm). Started on regular transfusion at 8 weekly intervals.
P11	M-9	Malay-Indian	Presented with anaemia (Hb 5.5 g/dL) at 6.5 years and given first transfusion, followed by 2 more in the same year. Patient remains asymptomatic and is able to maintain Hb between 7.4–9.5 g/dL without transfusion.
P12	M-13	Chinese	Presented with anaemia (Hb 5.8 g/dL) and hepatosplenomegaly at 2-month-old. First transfused at 4-month-old and transfused again at 7-month-old. Maintained on regular transfusion since 8-year-old to ensure normal growth and facies.
P13	M-9	Malay	Presented at 1-year-old with pallor and hepatosplenomegaly. Patient on hyper-transfusion regimen (4–6 weeks) due to poor growth.

P1: α-thalassaemia intermedia (α^codon 59^α/-α^3.7^).

P2–P12: Severe α-thalassaemia intermedia (α^codon 59^α/α^CS^α).

P13: Severe α-thalassaemia intermedia (α^codon 59^α/α^Paksé^α).

P7–P10^*^: Patients with one sibling confirmed with severe α-thalassaemia intermedia (α^codon 59^α/α^CS^α).
