# Supplementary material for: DNA studies are necessary for accurate patient diagnosis in compound heterozygosity for Hb Adana (HBA2:c.179>A) with deletional or nondeletional α-thalassaemia
Source: Sci Rep. 2016 Jun 8;6:26994. doi: 10.1038/srep26994 (PMC4897612; doi:10.1038/srep26994)

SUPPLEMENTARY INFORMATION

DNA studies is necessary for accurate patient diagnosis in compound heterozygosity for Hb Adana (HBA2:c.179>A) with deletional and nondeletional α-thalassaemia

Jin Ai Mary Anne Tan[1*], Siew Leng Kho[1], Chin Fang Ngim[2], Kek Heng Chua[1], Ai Sim Goh[3], Seoh Leng Yeoh[3] & Elizabeth George[4]

[1]Department of Biomedical Science, Faculty of Medicine, University of Malaya, Kuala Lumpur, Malaysia.

[2]Department of Paediatrics, School of Medicine and Health Sciences, Monash University Sunway Campus, Selangor, Malaysia.

[3]Department of Paediatrics, Hospital Pulau Pinang, Jalan Residensi, Penang, Malaysia.

[4]Assunta Hospital, Petaling Jaya, Selangor, Malaysia.

Table 1 Primer sequences for DNA amplification of five common α-thalassemia deletions: -$\alpha^{3.7}$, -$\alpha^{4.2}$, --^{SEA}, --^{FIL}, --^{THAI} using multiplex-PCR.

Deletion	Amplicon size (bp)	Forward primer sequence (5'–3')	Reverse primer sequence (5'–3')
-$\alpha^{3.7}$	2022	CCCCTCGCCAAGTCCACCC	AAAGCACTCTAGGGTCCAGCG
-$\alpha^{4.2}$	1628	GGTTTACCCATGTGGTGCCTC	CCCGTTGGATCTTCTCATTTCCC
--^{SEA}	1349	CGATCTGGGCTCTGTGTTCTC	AGCCCACGTTGTGTTCATGGC
--^{FIL}	1166	TGCAAATATGTTTCTCTCATTCTGTG	ATAACCTTTATCTGCCACATGTAGC
--^{THAI}	1024	GGCACTGAGAGCCCTTCACG	CAAGTGGGCTGAGCCCTTGAG

Table 2 Primer sequences for DNA amplification of six nondeletional α-thalassemia mutations characterised by Multiplex Amplification Refractory Mutation System (ARMS) – initiation codon (ATG→A-G), codon 30 (ΔGAG), codon 35(TCC→CCC), Hb Adana, Hb Constant Spring (HbCS) and Hb Quong Sze (HbQS).

Mutation	Amplicon size (bp)	Forward primer sequence (5'–3')	Reverse primer sequence (5'–3')
Initiation codon (ATG→A-G)	869	CACAGACTCAGAGAGAACCCAGCAG	TCTCCCCGCAGGATGTTCGTGC
Codon 30 (ΔGAG)	772	GTATGGTGCGGAGGCCCTGAG	TCTCCCCGCAGGATGTTCGTGC
Codon 35 (TCC→CCC)	645	TCTCCCCGCAGGATGTTCGTGC	TCTCCCCGCAGGATGTTCGTGC
Hb Adana	574	CTCTGCCCAGGTTAAGGGCCAAGA	TCTCCCCGCAGGATGTTCGTGC
HbCS	234	CCGTGCTGACCTCCAAATACGGTC	TCTCCCCGCAGGATGTTCGTGC
HbQS	184	CACCCCTGCGGTGCACGCCTCACC	TCTCCCCGCAGGATGTTCGTGC

Table 3 Primer sequences for DNA amplification of nondeletional α-thalassemia Hb Paksé and Hb Constant Spring (HbCS) using duplex-PCR.

Mutation	Amplicon size (bp)	Forward primer sequence (5'–3')	Reverse primer sequence (5'–3')
Hb Paksé	253	AGATGGCGCCTTCCTCTCAGG	ACGGCTACCGAGGCTCCAGCA
HbCS	180	GCTGACCTCCAAATACCGTC	CCATTGTTGGCACATTCCGG

Table 4 Primer sequences for DNA sequencing of α-globin gene.

Primer	Forward primer sequence (5'–3')	Reverse primer sequence (5'–3')
DNA sequencing primer	TGGAGGGTGGAGACGTCCTG	α2-globin gene: CCATTGTTGGCACATTCCGG

Figure 1A Electropherogram after DNA sequencing of genomic DNA from normal individual (top) and individual heterozygous for Hb Adana (lower) using forward sequencing primer. The lower electropherogram shows the G→A base substitution for Hb Adana

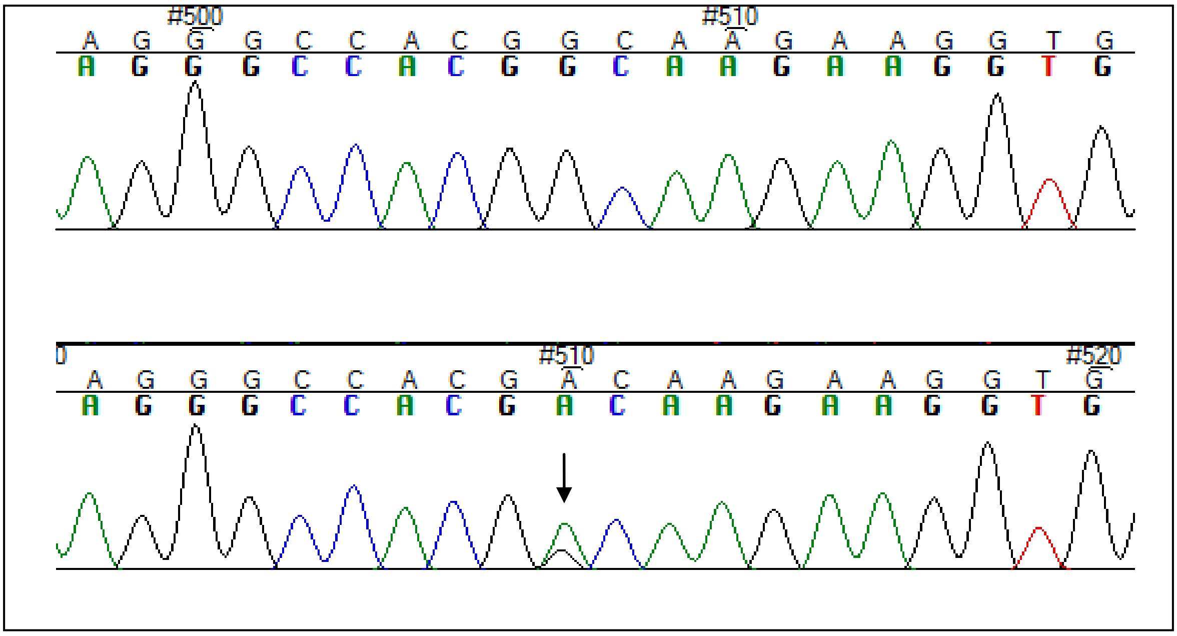

Figure 1B Electropherogram after DNA sequencing of genomic DNA from normal individual (top) and individual heterozygous for Hb Adana (lower) using reverse sequencing primer. The lower electropherogram shows the C→T base substitution for Hb Adana

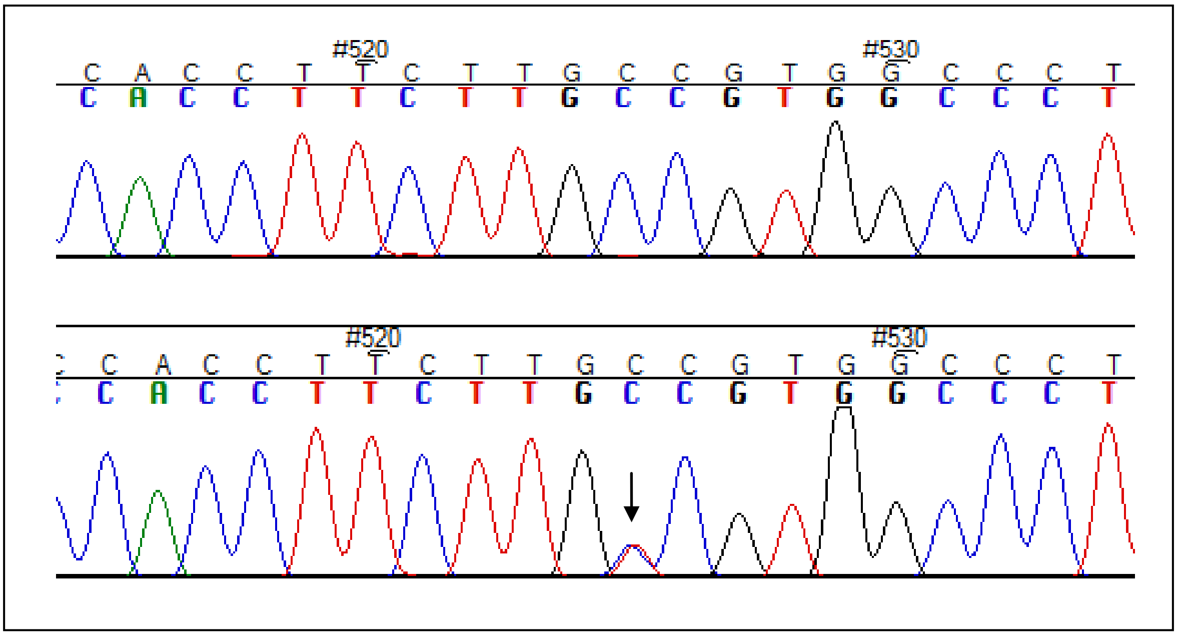

Supplement: Supplementary Information [file srep26994-s1.pdf]
